# Genetic Crossovers Are Predicted Accurately by the Computed Human Recombination Map

**DOI:** 10.1371/journal.pgen.1000831

**Published:** 2010-01-29

**Authors:** Pavel P. Khil, R. Daniel Camerini-Otero

**Affiliations:** Genetics and Biochemistry Branch, The National Institute of Diabetes and Digestive and Kidney Diseases, National Institutes of Health, Bethesda, Maryland, United States of America; University of Oxford, United Kingdom

## Abstract

Hotspots of meiotic recombination can change rapidly over time. This instability and the reported high level of inter-individual variation in meiotic recombination puts in question the accuracy of the calculated hotspot map, which is based on the summation of past genetic crossovers. To estimate the accuracy of the computed recombination rate map, we have mapped genetic crossovers to a median resolution of 70 Kb in 10 CEPH pedigrees. We then compared the positions of crossovers with the hotspots computed from HapMap data and performed extensive computer simulations to compare the observed distributions of crossovers with the distributions expected from the calculated recombination rate maps. Here we show that a population-averaged hotspot map computed from linkage disequilibrium data predicts well present-day genetic crossovers. We find that computed hotspot maps accurately estimate both the strength and the position of meiotic hotspots. An in-depth examination of not-predicted crossovers shows that they are preferentially located in regions where hotspots are found in other populations. In summary, we find that by combining several computed population-specific maps we can capture the variation in individual hotspots to generate a hotspot map that can predict almost all present-day genetic crossovers.

## Author Summary

In eukaryotes genetic crossovers are responsible for generating genetic diversity and ensuring the proper segregation of chromosomes. Genetic crossovers are tightly clustered in hotspots. Although the existence of hotspots in humans is clearly proven, mechanisms of their formation and the regulation of meiotic recombination in general remain poorly understood. An additional complication in studies of meiotic recombination is the fact that the direct experimental mapping of human hotspots on a genome-wide scale is not feasible with current methods. The best available indirect methods compute the position of hotspots from patterns of historic associations between genetic markers in population samples. In this study we determined the positions of genetic crossovers in ten pedigrees of European origin and then compared the positions of crossovers with the hotspots computed from HapMap data. Importantly, we find that the population-averaged computed map is in close agreement with the observed distribution of genetic crossovers. We also find that cryptic hotspots that are not easily detected in the computed European map can be more effectively identified if other populations are included in the analysis. Our analysis shows that high-resolution recombination profiles are highly similar between distantly related populations and that by including computed hotspots from several populations we can predict nearly all crossovers.

## Introduction

Meiotic crossovers are tightly clustered into hotspots in many organisms, including human, mouse and yeast [1]–[4]. Although hotspots may not be necessary to explain patterns of linkage disequilibrium in human populations [5], their existence is strongly supported by numerous experimental studies [6]–[9] (for review see [3],[4]) and detailed studies of the MHC class II region indicate that hotspots are mainly responsible for the patterns of linkage disequilibrium in that region [10]. High resolution experimental studies also show that hotspots are surrounded by regions of very low recombination rates, much lower than the genome average [8],[11],[12].

Although hotspots exist, their existence as well as their transmission from generation to generation is puzzling. According to current models of meiotic recombination [1]–[3] the fragment of DNA around the double strand break (DSB) from the initiating chromosome is replaced with the DNA sequence from the non-initiating chromosome. Therefore, if this initiating DSB is caused by a genetic element located inside or near the hotspot, theoretical studies predict that hotspots will self destruct (the hotspot paradox) [13]. As a consequence, theoretical analyses and computer simulations show that there should be a constant turnover of hotspots [14]–[16]. Thus, it is difficult to explain the existence and relative abundance of strong hotspots [16], although simulations suggest that genetic drift can lead to fixation of weaker hotspots [14],[15]. Some potential solutions of the hotspot paradox include alternative activation mechanisms [17],[18] or incorporation of natural selection in the analysis [19].

In agreement with theoretical analyses, a high level of variation in meiotic recombination has been observed in humans (for review see [4], [20]–[23]). It has been shown that there is essentially no correlation in the positions of the hotspots of meiotic recombination between chimpanzee and human in the roughly 1.5 Mb region compared [24]–[26]. In the shorter timescale of human evolution, variation in meiotic recombination between individuals and populations has been seen using both cytogenetic and genetic methods [23],[27],[28], by computational studies of patterns of linkage disequilibrium in several dozen human genes [29]–[35] and by the direct observation of polymorphisms in hotspots detected by sperm genotyping [7], [8], [11], [36]–[41]. Interestingly, on a megabase scale recombination rates appear to be similar between populations [42],[43] and even in distantly related species, such as human and mouse [44], suggesting the existence of constraints on domain-wide recombination rates.

The mechanisms responsible for hotspot formation and the regulation of hotspots in humans are poorly understood. If in yeast, for example, the presence or absence of short sequence motifs can turn on and off meiotic recombination at specific locations (see [2] for review); in mammals the situation appears to be more complex. Although a redundant 13-mer CCNCCNTNNCCNC has been identified in the human genome as associated with higher recombination rates [45] it is relatively non-specific and is found near only 40% of hotspots. Thus, it is unlikely that this motif can explain all hotspots in humans. Nevertheless, strand asymmetry in the initiation of meiotic recombination has been observed in several human [36],[37],[40],[41] and mouse hotspots [46]–[51]. It has been also shown that this haplotype-specific variation in meiotic recombination can be inherited [41]. This differential activity of haplotypes indicates that subtle sequence or epigenetic differences can dramatically influence meiotic recombination both at the level of hotspots and, as reported, on a more global level as well [27],[52]. In terms of the mechanism of regulation, both cis- and trans-activating genetic factors have been identified in human [53] and mouse [46],[49],[50]. Several chromatin modifications, notably H3K4 tri-methylation are associated with meiotic recombination in yeast [54],[55] and mouse [56]. A combination of such epigenetic and genetic factors is likely responsible for the high level of variation in meiotic recombination in humans.

Studies of the regulation of meiotic recombination are hampered by the fact that currently there is no practical way to experimentally determine genome-wide hotspot map in human. A commonly used approach suitable for defining genome-wide hotspot map is to calculate recombination rates from patterns of linkage disequilibrium in human populations. There are many potential reasons why the computed map may be different from the actual distribution of present-day crossovers. One possibility is rapid change in the meiotic hotspots. Another possibility is errors inherent in calculating recombination rate profiles from population variability data. The inaccuracy in defining recombination rates from sequence variation data is high [34], [57]–[59]. Current methods rely on a rather simplistic population history model and substantial deviations in local population history will affect rate estimates [58],[60]. Natural selection also may lead to both the “disappearance” [60] and “appearance” [61] (although these findings were later disputed [62]) of hotspots. A lack of diversity in population samples will also lead to an inability to accurately reconstruct recombination rates (see [60] and references therein). Thus, it is important to establish how well the computed map predicts genetic crossovers.

Recently Coop et al. [52] performed a genome-wide mapping of meiotic crossovers in Hutterites and compared the locations of crossovers with the positions of computed hotspots. They reported that while the majority of crossovers originate in hotspots, approximately 40% of recombination events take place outside of hotspots calculated from patterns of linkage disequilibrium. Moreover, they observed a great variation in the usage of LD-defined hotspots in different individuals. In up to a third of individuals the estimated hotspot usage (fraction of recombination events that originate in hotspots) is below 50% and even reaches 0% in two individuals (95% confidence interval less than 50% usage). This observation suggests that the calculated map may not accurately describe the distribution of meiotic recombination events in some individuals.

We, however, believe that some of the calculations in the Coop et al. paper are may not accurately reflect the similarity of the crossover distribution to the computed map. The most important conclusions in the Coop paper are based on the use of an indirect estimate for the true fraction of crossovers that originate in hotspots that did not take into account differences in hotspot strength and the variation in the background recombination rate. All calculations are based on “hotspot usage” as defined by Coop et al. This usage, however, would be identical whether a very strong hotspot is surrounded by areas of low recombination rate or a very weak hotspot is in a region with a high background. To evaluate the accuracy of the computed map we have mapped crossovers in CEPH pedigrees and then estimated how well this map predicts the positions of the crossovers. In our work we are not only asking if hotspots explain all crossovers, but also if the distribution of crossovers is consistent with the computed map.

## Results

### Mapping crossovers in CEPH families

To define regions recombining in the present day we determined 4778 intervals containing crossovers in 69 siblings from ten large CEPH Utah reference families (CEPH/UTAH Pedigrees 1334, 1340, 1341, 1350, 1362, 1408, 1420, 1447, 1454 and 1459, grandparents and parents from these families were previously genotyped by the HapMap project [32],[63] as a part of the CEU population) using the Affymetrix 500K mapping set (see Methods). To map crossover positions from SNP genotype data we developed an algorithm that phases chromosomes in nuclear families with multiple siblings and then determines regions where derived chromosome sequence switches from one of the parental chromosomes to the other. We first determined phase in the positions where trivial haplotype inference is possible (SNPs homozygous in one parent and heterozygous in the other) and then in the positions heterozygous in both parents (for details see Text S1). The uncertainty in defining crossover positions ranges from 50 bp to over 30 Mb (a crossover mapped to centromere of chromosome 9) with a median of ∼70 Kb (Figure S1, Table S1, Table S2). The patterns of the distribution of crossovers such as an excess of maternal crossovers, and telomeric distribution of paternal crossovers are consistent with previously reported observations (Figure S2). We achieved substantially higher resolution of crossover mapping (70 vs 93 Kb) than has been reported before [52], although it is not clear whether this improvement is due to the more precise crossover mapping or results from differences between the CEPH and Hutterite datasets. The higher resolution of crossover mapping may be partially explained by the ∼10% higher number of genotyped SNPs in our study and by the larger number of children per CEPH family (6.9 on average) compared to the number of children per Hutterite family.

We used a coalescence-based computational approach [57] (see Methods for details on computational procedures) to estimate the genome-wide recombination rates for each of the populations represented in Phase II of the HapMap dataset [32] and then we identified hotspots in each of the population-specific recombination rate maps and in a population-averaged map (Figure S3, Table S3). Hotspots were defined as peaks in the recombination rate profile less than 100 Kb in width with strength above 0.01 cM. The use of this definition results in the identification of 45,872 hotspots in the CEU sample (see Text S1 for details). In addition to using the peak-based definition of the hotspots, we also included in the analysis 32,996 hotspots previously inferred from the HapMap Phase II dataset using the likelihood-ratio test implemented in LDHot [32],[63]. LDHot hotspots were defined as hotspots detected in more than one population and thus they are not population specific. The work by Coop et al [52] is based exclusively on LDHot hotspots and did not take into account differences in the strengths of the hotspots.

### The population-averaged map accurately describes the distribution of present day crossovers

**Figure 1:**
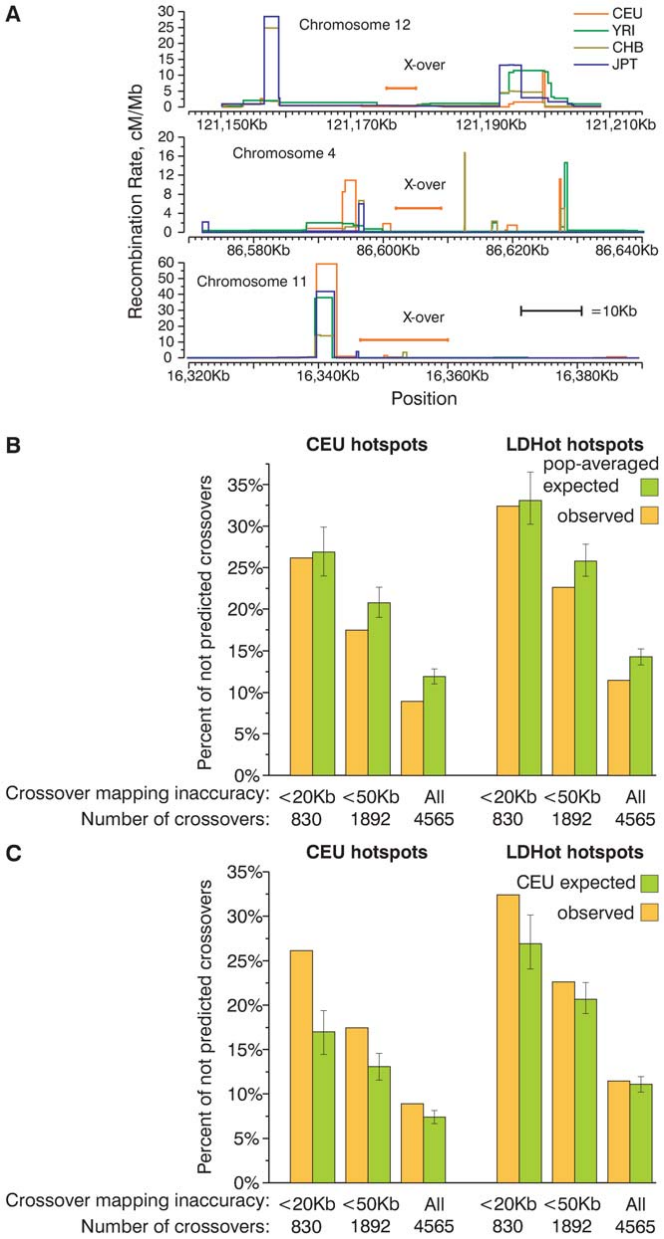
A substantial fraction of present-day crossovers is not predicted by historic recombination rate profiles. (A) Examples of small present day crossover intervals that do not overlap historic hotspots. (B,C) Percentage of present-day crossovers in CEPH families not predicted by overlapping hotspots. The percentages of crossovers that do not overlap CEU and LDHot hotspots were calculated for several subsets of all crossovers defined with various degrees of accuracy. For comparison, the same percentage was calculated for crossovers distributed according to probabilities determined by population-averaged (B) and CEU (C) recombination rate maps. Mean and 95% CI are plotted on the graph. A large fraction of crossovers is not predicted by hotspots.

First we asked how well hotspots predict CEPH crossovers. Since the average size of crossover-containing intervals is comparable with the distance between hotspots, some crossover intervals overlap hotspots due to our inability to map them precisely. To address this issue we analyzed separately three subsets of crossovers mapped to intervals of different size (Figure 1). Smaller crossover intervals are less likely to overlap hotspots by chance (Figure S4).

In general, the distribution of present-day crossovers is clearly non-random. Crossovers are relatively well predicted by either LDHot- or peak-defined hotspots. The majority of crossover intervals overlap hotspots and the proportion of predicted crossovers is significantly higher (P<0.001 by simulation) than for identically sized crossover intervals randomly distributed in the genome. While 68%–74% of crossover intervals smaller than 20 Kb overlap hotspots, only 22 and 30% of randomly distributed crossovers intervals are expected to overlap hotspots (Figure S5, for details on simulation see Text S1). Nevertheless, we find that 26% of the present-day crossover intervals smaller than 20 Kb do not overlap CEU hotspots and 32% of crossover intervals do not overlap LDHot hotspots (Figure 1B and 1C). As expected, the percentage of crossover intervals overlapping hotspots is dependent on how accurately we can map the crossovers (Figure 1B and 1C, Figure S4). The percentage of not predicted (we consider crossovers to be “predicted” if crossover intervals overlap at least one hotspot) crossovers in the CEPH sample is very close to the percentage of not predicted crossovers previously reported in Hutterites (28% for crossover intervals smaller than 30 Kb [52] and Figure S6).

Hotspots account for only 71–79% of the genetic map (Table S3). Thus, even if crossovers would be distributed in perfect agreement with the map, a fraction of crossovers proportional to the fraction of the recombination rate map that lies outside hotspots is expected to be not predicted by hotspots. An additional complication in estimating the expected fraction of crossovers that overlap hotspots by chance arises from the limited resolution of the mapping of crossovers. The percentage of crossovers predicted by chance depends on the size and distribution of hotspots and the size of the crossover intervals. To calculate the expected fraction of predicted crossovers we performed a computer simulation. We re-distributed the experimentally determined crossover intervals according to the computed recombination rate map (Text S1). We generated 1000 datasets where crossovers were distributed according to the CEU or the population-averaged maps (Figure 1B and 1C).

Both the CEU and LDHot hotspots predict at least as many as expected CEPH crossovers (Figure 1B, Figure S6) and Hutterite crossovers (Figure S7) if we re-distribute crossovers according to the population-averaged map. For both sets of hotspots the fraction of not predicted crossovers is significantly lower than expected for crossover intervals smaller than 50 Kb and all crossover intervals (P<0.001 by simulation, Figure 1B, Figure S7, S8) and not significantly different for crossover intervals smaller than 20 Kb (P = 0.34 for CEU hotspots, P = 0.37 for LDHot hotspots, Figure 1B, Figure S7, S8). Thus, the observed fraction of not predicted crossovers agrees with the expected fraction of not predicted crossovers if crossovers are distributed according to the population-averaged map.

Unlike the results for the simulation with the population-averaged map, we find that when crossovers are distributed according to the CEU map the fraction of crossovers that are not predicted by the CEU hotspots is significantly higher than expected (P<0.001 by simulation for all sets of crossovers, Figure 1C, Figure S8). For LDHot hotspots, the fraction of not predicted crossovers is significantly higher than expected for crossover intervals smaller than 20 Kb and 50 Kb (P<0.001, P<0.017 by simulation, respectively) and is not different from expectations if we compare all crossovers (P = 0.18) (Figure 1C, Figure S7). This excess of not predicted crossovers is observed for all subsets of Hutterite crossovers as well (Figure S7). Thus, our computer simulation is sensitive enough to distinguish the population-averaged map from the CEU map and the population-averaged map appears to be closer to the observed distribution of crossovers.

### Hotspots of different strengths are detected in the computed map with comparable efficiency

**Figure 2:**
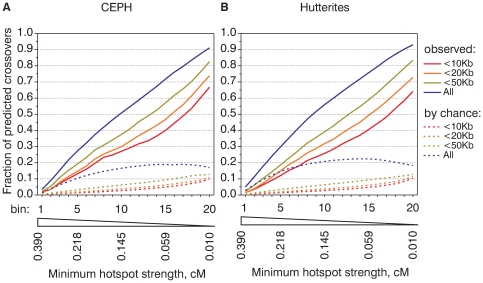
Hotspots of different strengths are equally active in recombination. Cumulative recombination frequency graphs of CEPH (A) and Hutterite (B) crossovers. All CEU hotspots were ranked by the strength from the strongest (bin1) to weakest (bin20) and divided into twenty bins of equal aggregate strength. For each bin we calculated fractions of CEPH (A) and Hutterite (B) crossovers predicted by hotspots from analyzed bin and bins with stronger hotspots. For the analysis all crossovers were divided in sets based on the mapping accuracy. For comparison, we calculated the fractions of crossovers that overlap hotspots by chance (see Text S1 for details on calculation) and plotted their mean values (dashed lines).

The comparison of observed and expected fractions of predicted crossover intervals did not take into account the relative strength of individual hotspots. One can imagine that weak or strong hotspots are predicted with different efficiency. To estimate the relative impact of hotspots of different strength on present-day crossovers we calculated how frequently hotspots of different strength overlap crossover intervals. Because the number of hotspots in the human genome is larger than the number of mapped crossovers in either CEPH or Hutterite datasets, we cannot perform such an assessment for the majority of individual hotspots. To account for this relatively low number of crossovers we grouped together hotspots of similar strengths. We ranked all CEU hotspots based on their strength and divided them into twenty bins of equal aggregate strength, so each of the bins is expected to predict an equal fraction of crossovers. For example, the first bin contains the 261 strongest hotspots and the last bin (bin number 20) contains the 11,837 weakest hotspots, but both are expected to predict 5% of crossovers (Table S4). We then calculated the percentage of crossovers actually predicted by each of the bins and plotted these values (Figure 2 and plotted according to the minimal strength of the hotspots in the bin in Figure S9, similar analysis performed for LDHot hotspots is presented in Figure S10). This cumulative recombination frequency graph indicates the relative capacity in predicting crossovers of hotspots of different strengths.

In the ideal case if crossovers could be mapped precisely, if hotspots could explain all crossovers and if the strength of hotspots could be estimated without errors we would expect to see a straight diagonal line with exactly 5% of crossovers per bin. The observed shape of the cumulative frequency graph although not ideal, is reasonably close to a straight line. This indicates approximately equal contributions from hotspots of different strength. The cumulative recombination frequency graphs are highly similar for the CEPH and Hutterite datasets (Figure 2) and for LDHot-defined hotspots (Figure S10). On the other hand, there is a marked difference in the slope of the cumulative frequency graph for subsets of crossovers mapped to larger and smaller intervals. This difference in the slope cannot be completely accounted by crossovers that overlap hotspots by chance (see Figure 2). This is likely an expression of finer differences between the computed map and the observed distribution of crossovers and indicates a tendency for not predicted crossovers to locate near hotspots.

### The observed cumulative recombination frequency graphs are similar to those expected from the population-averaged map

**Figure 3:**
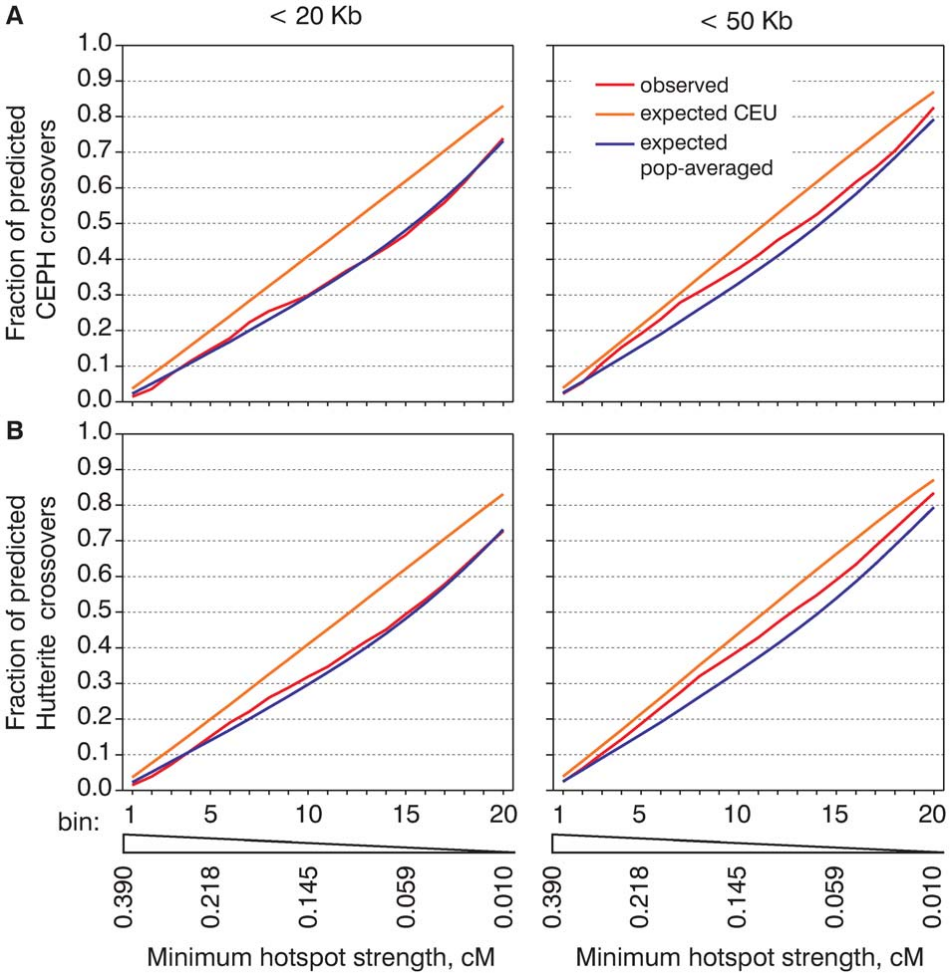
The population averaged map is much closer to the distribution of meiotic crossovers than the CEU map. We calculated and plotted cumulative recombination frequency graphs for CEPH (A) and Hutterite (B) crossovers and the cumulative recombination frequency graphs for crossovers re-distributed according to the population-averaged or CEU maps.

Compared to the analysis presented on Figure 1 where all of the hotspots were combined, the cumulative recombination frequency graphs reflect the relative activity of hotspots of different strengths. To better estimate how close the computed recombination rate maps are to the observed distribution of crossovers we compared the observed cumulative recombination frequency graphs with those obtained by computer simulation (Figure 3, Figure S11). Here we again clearly see that the crossover distribution both in CEPH and Hutterite datasets resembles the population-averaged map better than the CEU map. We must note, however, that the observed distribution of crossovers is not identical to that of either the CEU or the population-averaged map. For most subsets of crossovers hotspots predict more crossover intervals than expected from the population-averaged map. This suggests that the population-averaged map slightly underestimates the strength of hotspots and the peak rate inside them. For the CEU map we see exactly the opposite effect — hotspots predict less crossovers than expected. This means that the CEU map tends to overestimate the strength of some hotspots and that the actual distribution of crossovers is less concentrated in hotspots compared to what would be expected from the CEU map.

### How many crossovers are not predicted by hotspots?

Because some crossovers can overlap hotspots by chance, the observed proportion of crossover intervals overlapping hotspots can be higher than the true fraction of crossovers that were initiated in hotspots. There are several ways to estimate the proportion of hotspot-derived crossovers. One way is to calculate the fraction of predicted crossovers assuming that the distribution of not predicted crossovers is known. We have uniformly re-distributed crossovers near their original location and Coop et al [52] re-distributed crossovers normally. An application of this approach results in estimates of 23%–33% for the fraction of not predicted crossovers for smaller and larger crossover intervals respectively (see Text S1, Figure S12, Table S5 for details).

Another way to estimate the true proportion of hotspot-derived crossovers comes from examining cumulative frequency graphs. In the ideal situation for a perfect correlation between the map and the observed distribution of crossovers each bin would predict exactly 5% of crossovers. The difference between the “ideal” 5% slope and the observed slope in the cumulative recombination frequency graph is an estimate of the proportion of not predicted crossovers. This estimate is based on two assumptions: that in the middle of graph the fraction of crossovers overlapping hotspots by chance is low and that hotspots from all of the bins are equally effective in initiating crossovers. The first assumption is justified by the relatively low number of hotspots in the “stronger” bins. The fraction of crossovers that overlap hotspots by chance depends on the number of hotspots. The total number of hotspots in the first ten bins is only 7,778, or approximately 1/6 of all hotspots. We estimate that less than 0.5% crossovers per bin overlap hotspots by chance (see Figure 2). The second assumption is justified by the relatively linear shape of the graph.

The slope in the middle of the cumulative frequency graph is between 0.034/bin and 0.042/bin for the smaller and larger crossover intervals respectively, resulting in estimates of the not predicted fraction of between 0.016 and 0.008 per bin or, if we extend this estimate to all twenty bins we obtain 16–32% for all hotspots. Thus, application of both approaches results in similar estimates of 16–33% for the fraction of not predicted crossovers.

### Which hotspots are best in predicting CEPH crossovers?

**Figure 4:**
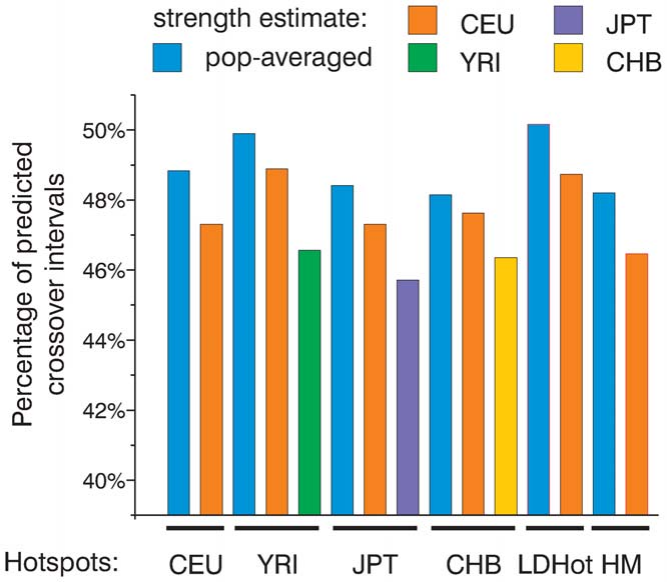
All sets of hotspots are similarly efficient in predicting crossovers. We calculated and plotted the fraction of crossover intervals smaller than 50 Kb overlapping 10,000 strongest hotspots defined in several ways. We have used LDHot hotspots (LDHot) and peak-based hotspots from the population-averaged map (HM) and four population specific maps (CEU, YRI, JPT, and CHB). For ranking, we have used either four population-specific or population-averaged strength estimates.

So far we have shown that the observed distribution of CEPH crossovers closely resembles the distribution expected from the population-averaged map. An independent question is which set of hotspots is best at predicting crossovers. We have four populations-specific sets of peak-defined hotspots, the population-averaged set of peak-defined hotspots and LDHot hotspots. To compare these six independent hotspot sets we again ranked hotspots based on their strength calculated from either one of the population-specific or the population-averaged maps. We then took the 10,000 strongest hotspots and compared the numbers of crossovers overlapping them (Figure 4). First of all, all the sets of hotspots have a very similar efficiency in predicting crossovers. These 10,000 strongest hotspots overlap between 46% and 50% of crossover intervals smaller than 50 Kb. When we use either population-specific or population-averaged recombination rate estimates for ranking, the 10,000 strongest hotspots according to the population-averaged map always predict more crossovers (Figure 4). This again proves that the population-averaged map is closer to the actual distribution of crossovers and provides the best estimate of hotspot strengths. Out of all sets, the LDHot-defined hotspots overlap the largest number of crossover intervals (50.2%). Thus, LDHot-defined hotspots are most efficient in identifying universally conserved, strongest hotspots. When we compare observations to expectations, hotspots predict crossovers better than expected from the population-averaged map (Figure S13). Along with our previous comparison of cumulative recombination frequency graphs this observation again suggests that the peak rate in hotspots is slightly underestimated.

### Where are the not predicted crossovers?

We find that 26–32% of crossovers cannot be explained by either CEU hotspots or LDHot-defined hotspots, respectively. First we asked if there is one or several large genomic regions where the distribution of crossovers strongly deviates from the hotspot map. An examination of the genomic distribution of not predicted crossover on a large scale does not show a strong tendency towards accumulation in specific genomic region(s) (Figure S14). Thus, it is unlikely that all not predicted crossovers can be explained by such local deviations.

**Figure 5:**
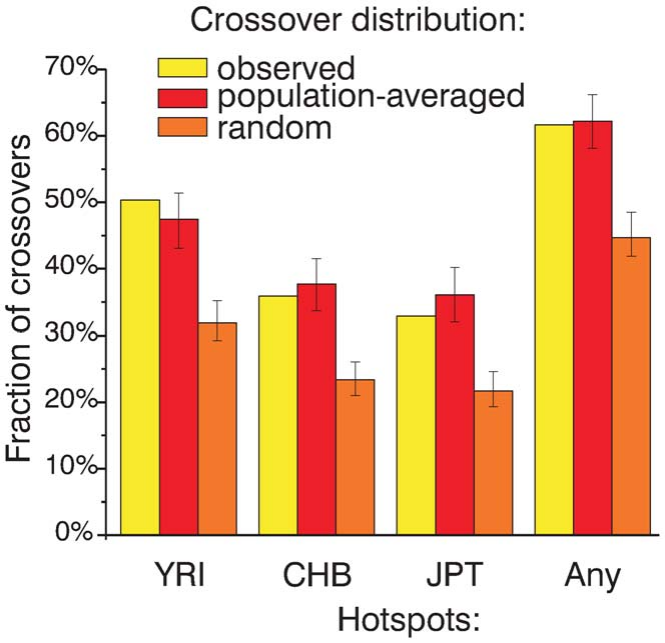
Most CEPH crossovers not predicted by CEU hotspots overlap hotspots from other populations. The fraction of crossover intervals not predicted by CEU hotspots that overlap hotspots found in YRI, CHB, JPT, or any of the other HapMap PhaseII populations (YRI, CHB, or JPT) is plotted. For comparison, the same fraction of crossovers overlapping hotspots from other populations (mean and 95% CI) is plotted for crossovers re-distributed according to the population-averaged map and randomly distributed crossovers.

The finding that the population-averaged map is in closer agreement with the distribution of crossovers compared to the CEU map suggests that hotspots from other populations may be in fact active in the CEU sample but not detected in the CEU profile. Thus, we asked where such not predicted crossovers are located relative to hotspots detected in other populations. We find that, depending on the accuracy of mapping, between 50 and 61 percent of not predicted crossovers overlap at least one hotspot from another population (YRI, CHB or JPT) (results for CEPH crossovers are shown in Figure 5 and for Hutterite crossovers are shown in Figure S15). Importantly, this proportion is significantly higher than expected if crossovers would be distributed randomly (P<0.001 by simulation, see Figure 5 and Figure S15 for all crossovers; data are not shown for other subsets of crossovers) meaning that crossovers are preferentially located in regions where hotspots are found in other populations. Furthermore, as one might expect, the fraction of not predicted crossovers that overlap at least one hotspot from another population is similar to the expected proportion if crossovers would be distributed according to the population-averaged map (Figure 5, Figure S15).

A potential explanation for the preferential co-localization of crossovers not predicted by CEU hotspots with the hotspots from the other populations could be the increased power to detect hotspots in the larger combined population set. To check whether adding individuals from the same population sample increases our ability to predict crossovers as well as adding individuals from a different population we generated 100 subsets from the CEU and CHB samples and then calculated hotspots as for the full samples. We find that hotspots from subsets of the CHB sample overlap more not predicted crossovers compared to hotspots from matching subsets of the CEU sample (Figure S16). A lack of SNP diversity in population sample decreases the power of LD-based coalescent analysis to accurately estimate recombination rates. Thus, one interpretation of the more frequent association of not predicted crossovers with the hotspots found in other populations is a lack of SNP diversity in the CEU sample. To evaluate the effect of the SNP diversity on identification of weaker hotspots we compared average minor allele frequencies in the four population samples in predicted and not predicted crossover intervals smaller than 50 Kb. (Figure S17). The average minor allele frequency (MAF) for SNPs located inside not predicted crossover intervals is not lower than the MAF in predicted crossovers. Thus, other underlying differences are likely responsible for the preferential association of the not predicted crossovers with the hotspots found in other populations.

## Discussion

In this work we analyze the distribution of meiotic crossovers mapped at high resolution and use this dataset to probe calculated maps. Our main conclusion is that the calculated recombination rate map is in good agreement with observations. Although we estimate that more than 30% of crossovers are not predicted by hotspots, a number in agreement with previous findings [52], this does not necessarily mean that the distribution of crossovers is different from the computed map. We calculate that if crossovers are distributed according to the population-averaged map we expect to find approximately as many not predicted crossovers, roughly one third, as we estimate from crossover mapping data. Moreover, the inclusion of hotspots from other populations allows us to account for the majority of not predicted crossovers.

Coop et al. [52] reported less that 50% usage of LDHot hotspots in roughly 30% of Hutterites. Taken by itself, this observation could suggest relatively poor agreement between LDHot hotspots and positions of crossovers in Hutterites. While Coop et al. have asked whether all crossovers overlap hotspots we addressed an arguably more relevant question whether the observed distribution of crossovers is consistent with the recombination rate map. LDHot hotspot usage utilized by Coop et al. to describe the similarity of recombination map to observations is an indirect estimate of true proportion of recombination events in hotspots. That analysis considered only the location of hotspots and did not take into account the non-uniformity of recombination rates. When we carefully account for recombination rate variation in the computed map, we don't observe a strong disagreement between the positions of genetic crossovers and computed hotspots. Both our analysis of the CEPH dataset and our independent re-analysis of the Hutterite data suggests that all crossovers taken together agree with the computed map. This does not mean that there are no individuals with substantial differences in hotspots use. We would argue, however, that individuals in which meiotic crossovers occur mostly outside of hotspots are relatively rare, at least in European populations. Moreover, we observe a great degree of similarity in the ability of population-specific hotspots to predict both CEPH and Hutterite crossovers suggesting that a much lower than average hotspot usage is rare in all populations.

Although 30% of crossovers are not predicted by hotspots, we believe that this fact is largely a reflection of the properties of the computed map itself and hotspot definition rather than a measure of the dis-similarity of the crossover distribution to the map. Neither peak-based nor LDHot hotspots account for more than 79% of the total genetic map length. So, most not predicted crossovers can be accounted for by this “outside of the hotspots” part of the map. Why is not all of the genetic map captured by hotspots? Does it mean that not all recombination events occur in hotspots? Although this question is difficult to address directly based on crossover mapping data, the preferential location of not predicted crossovers where hotspots are found in other populations suggests otherwise. Multiple sperm genotyping studies show very low levels of background, non-hotspot recombination [7], [12], [36]–[38]. It is likely that weaker and difficult to detect hotspots are responsible for most of the not predicted crossovers. Computational methods are not sufficiently sensitive to detect these weaker and/or polymorphic hotspots. Difference in population sample histories and random errors in the estimation of recombination rates may result in a more efficient detection of some weak hotspots in other populations. It is also possible that these undetected hotspots are stronger in other populations.

Our analysis shows that in addition to hotspot position, our computed estimates of hotspot strength are largely accurate. One consequence of that is that both very strong and very weak hotspots exist. For example, the 700 strongest hotspots (Bins 1 and 2, mean strength 0.41 cM and representing 10% of the total hotspot strengths) account for 9% of all CEPH crossovers (Figure 2). Even if we conservatively estimate that half of these 9% of all CEPH crossover intervals overlap these 700 hotspots by chance, we still find support for more than several hundred hotspots stronger than 0.2 cM. Thus, in agreement with observations by Jeffereys [40] and Coop et al. [52] we find that very strong hotspots do exist. A similar logic supports the existence of weak hotspots. Bin 20 which contains more than 10000 hotspots between 0.01 and 0.016 cM accounts for 4–6% of crossovers.

**Figure 6:**
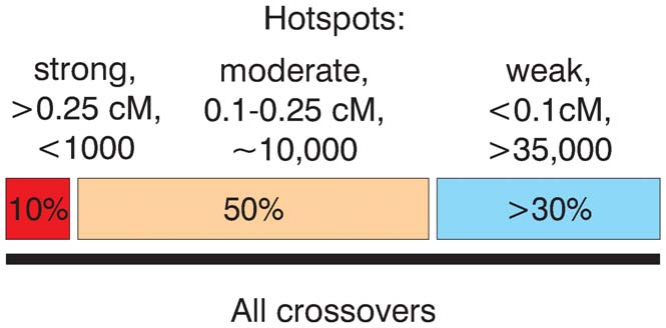
Schematic representation of the relative input of strong and weak hotspots to the total set of crossovers.

Then, how many hotspots of meiotic recombination exist in humans? This number clearly depends on how hotspots are defined. The application of the rather conservative LDHot method to the HapMap Phase II dataset results in the identification of nearly 33,000 hotspots [32],[63]. If we look simply for peaks in the recombination rate profile, we find around 50,000 peaks with an estimated strength above 0.01 cM or more than 150,000 peaks if we don't restrict hotspot strength. Our probing of the calculated map with present day crossovers gives some further insight into this question (Figure 6). There are several hundred, perhaps up to a thousand strong hotspots (calculated strength above 0.25 cM), but it is unlikely that they are responsible for more than 10% of all crossovers. Around 50% of crossovers (see Figure 2 and Figure 4) of crossovers is explained by roughly 10000 moderately strong hotspots between 0.1 and 0.25 cM. Then, there are tens of thousands of weak and/or polymorphic hotspots. Although individual hotspots are weak, more than 30% of all crossovers are explained by hotspots weaker than 0.1 cM. We also believe that the remaining 10% or so unaccounted for crossovers (see below) are largely due to cryptic hotspots. It is likely that the number of such cryptic hotspots is not smaller than the number of the detected “weak” hotspots, roughly 35,000. We estimate, therefore, that the total number of active hotspots, including polymorphic ones may reach up to 60,000–80,000 or more.

What are the implications of our study for the mechanism of hotspot variability within a population and between populations? We find that inclusion of hotspots from other populations allows us to account for 61% of crossovers (here we consider the set of all crossovers not restricted by the accuracy of mapping) that do not overlap CEU hotspots. In total, 95–97% of all of the observed crossovers can be accounted for by the hotspots from all four populations. Also, a comparison of different population-specific hotspots shows that all of them are highly efficient in predicting CEPH crossovers. Similarly, the better agreement between the distribution of CEPH crossovers and the population-averaged map rather than with the CEU map argues that there is a greater similarity between the recombination rate maps of different populations than what we are able to compute at this time. One way to reconcile these data is that hotspots arise at a limited number of potential sites. Consequently, different populations have hotspots mostly at the same locations although their strengths vary. The site selection for meiotic DSB formation is driven by a genomic susceptibility profile, defined either by nucleotide sequence or by chromatin structure, which determines propensity to form meiotic DSBs at a given location, a universal recombinome for humans. This susceptibility profile results in a set of potential hotspots which are sampled in different individuals and populations and is further regulated at a higher, perhaps domain-wide level. In yeast, for example, chromatin modifications have a profound effect on meiotic recombination [54],[55] and trans-activating regulators has been described in mammals [46],[49],[50],[53]. We also suggest that this intrinsic genomic susceptibility profile is largely intact between populations and individuals and most of the variation is seen at the level of the strength of the hotspots. This situation is very much akin to the variability in levels of gene expression in different individuals [64]–[66]. Variation in gene expression is caused both by genetic and epigenetic factors and is heritable to a large extent [65],[66]. As with the recombinome, all genes are present in all individuals but the level of transcripts is highly variable among individuals.

What are practical implications of our analysis? First of all, we find that the computed recombination rate map closely approximates present-day recombination profiles. Second, we find that it is important to include in the analysis samples from distantly related population samples. Both the closer similarity of the population-averaged profile to observations and the frequent detection of cryptic hotspots in other population-specific profiles clearly show that meiotic recombination in present-day individuals of European descent is better described by looking at more than one HapMap population. Presently and in the nearest future the experimental determination of individual recombination rate maps is still beyond our capabilities. We believe that the increased availability of high resolution data from diverse population samples, such as the ongoing Phase III of HapMap project, will allow highly accurate computational reconstruction and will provide further insights into hotspot variability and the regulation of meiotic recombination.

## Methods

### Recombination rate calculations

To calculate recombination rates we used LDHat version 2 [57] with minor modifications. We have used the complete Phase II data (phased genotypes from release 21a) from the HapMap project as a source of genotypes (www.hapmap.org and [32],[63]). Hotspots were defined as relatively narrow peaks (peak width <100 Kb) having strength above 0.01 cM. All coordinates are given relative to the NCBI35 version of the human genome assembly. Statistical calculations were performed in JMP version 7. This study utilized the high-performance computational capabilities of the Biowulf Linux cluster at the National Institutes of Health, Bethesda, MD (http://biowulf.nih.gov).

### Crossover mapping

DNA samples from the CEPH/UTAH pedigrees 1334, 1340, 1341, 1350, 1362, 1408, 1420, 1447, 1454 and 1459 were obtained from the Coriell cell repository. Samples were genotyped using Affymetrix 500K genotyping array sets according to recommendations of the manufacturer. To map crossovers we developed a multi-step algorithm (see Text S1) based on mendelian inheritance.

## Supporting Information

Figure S1 Histogram of the distribution of sizes of crossover intervals mapped in CEPH pedigrees. Summary statistics of the distribution are shown on the right.
(0.55 MB TIF)

Figure S2 Present-day crossovers of paternal origin are preferentially located in telomeric regions. The frequency of crossovers in bins uniformly distributed along chromosome length is shown on the graph. To allow cross-comparison of different chromosomes the positions of individual crossovers relative to chromosomes where they reside are shown in normalized chromosome units. Chromosome units were defined as the distance to the crossover from the short arm terminus divided by the corresponding chromosome length. There is an excess of paternal crossovers in telomeric regions but maternal crossovers are distributed relatively uniformly. There is also 59% excess of maternal crossovers over paternal crossovers (2,934 maternal crossovers compared to 1,844 paternal crossovers).
(0.53 MB TIF)

Figure S3 Summary information on hotspot maps in four HapMap Phase II samples. Histograms of the distributions of the hotspot strength, inter-hotspot distance and hotspot width are shown for CEU (A), YRI (B), CHB (C), and JPT (D) samples. In addition, the figure shows quantiles and mean values calculated for the corresponding distributions.
(0.05 MB PDF)

Figure S4 Proportion of randomly distributed crossover intervals that overlap hotspots depends on the size of crossover interval. The percentage of the crossover intervals overlapping CEU and LDHot-defined hotspots is plotted against the size of intervals. The percentage is averaged over 1,000 randomly generated samples.
(0.22 MB TIF)

Figure S5 Hotspots predict much larger fraction of present-day crossovers than expected by chance. Percentage of present-day crossovers in CEPH (A) and Hutterite (B) families not predicted by overlapping hotspots. The percentages of crossovers that do not overlap CEU and LDHot hotspots were calculated for three subsets of all crossovers defined with various degrees of accuracy. For comparison, the same percentage was calculated for randomly distributed crossovers (see Text S1). Mean and 95% CI are plotted on the graph.
(0.55 MB TIF)

Figure S6 Detailed simulation of crossover mapping confirms that the distribution of CEPH crossovers agrees with population-averaged recombination rate map. The percentages of CEPH crossovers that do not overlap CEU and LDHot hotspots were calculated for several subsets of all crossovers defined with various degrees of accuracy. For comparison, the same percentage was calculated for crossovers distributed according to probabilities determined by population-averaged recombination rate maps. Mean and 95% CI are plotted on the graph. In this analysis we simulated whole crossover detection and downstream analysis as close as possible to crossover mapping in CEPH families. We first re-distributed all CEPH crossovers according to the population-averaged map and then generated genotypes containing crossovers at defined positions. We then mapped crossovers using our algorithm.
(0.25 MB TIF)

Figure S7 A substantial fraction of present-day crossovers is not predicted by historic recombination rate profiles. (A,B) Percentage of present-day crossovers in Hutterite families not predicted by overlapping hotspots from the CEU profile. The percentages of crossovers that do not overlap CEU and LDHot hotspots were calculated for several subsets of all crossovers defined with various degrees of accuracy. For comparison, the same percentage was calculated for crossovers distributed according to probabilities determined by CEU (A) and population-averaged (B) recombination rate maps. Mean and 95% CI are plotted on the graph. A large fraction of crossovers is not predicted by hotspots.
(0.48 MB TIF)

Figure S8 Estimation of the statistical significance of the differences between observed and expected numbers of predicted crossovers. Hotspots predict a significantly smaller number of CEPH crossovers than expected from CEU map (A) and significantly larger number of CEPH crossovers than expected from population-averaged map (B). On the graph the histograms of the expected numbers of crossovers overlapping CEU and LDHot hotspots are plotted (1,000 samples) for three subsets of the crossovers (defined as in text before). For the estimation of expected numbers of predicted crossovers we randomized positions of crossover intervals in the genome according to probabilities determined by CEU (A) and population-averaged (B) recombination rates. The observed numbers of crossovers overlapping CEU or LDHot hotspots for the crossovers mapped in CEPH pedigrees are shown by arrows. The one-sided probability of finding the observed number or fewer of randomly distributed crossovers predicted by hotspots is in the range from 0.001 to 0.20 for crossovers distributed according to CEU map. The one-sided probability of finding the observed number or more of randomly distributed crossovers predicted by hotspots is less than 0.001 for two larger subsets of crossovers distributed according to population-averaged map.
(0.03 MB PDF)

Figure S9 Hotspots of different strengths are equally active in recombination. Cumulative recombination frequency graphs of CEPH (A) and Hutterite (B) crossovers. All hotspots were ranked by the strength from the strongest (bin1) to weakest (bin20) and divided into twenty bins of equal aggregate strength. For each bin we calculated fractions of CEPH (A) and Hutterite (B) crossovers predicted by hotspots from analyzed bin and bins with stronger hotspots and plotted this fraction against the minimum hotspot strength from the analyzed bin. For the analysis all crossovers were divided in sets based on the mapping accuracy.
(0.40 MB TIF)

Figure S10 Hotspots of different strengths are equally active in recombination. Cumulative recombination frequency graphs of CEPH (A) and Hutterite (B) crossovers. All LDHot-defined hotspots were ranked by the strength from the strongest (bin1) to weakest (bin20) and divided into twenty bins of equal aggregate strength. For each bin we calculated fractions of CEPH (A) and Hutterite (B) crossovers predicted by hotspots from analyzed bin and bins with stronger hotspots. For the analysis all crossovers were divided in sets based on the mapping accuracy.
(0.45 MB TIF)

Figure S11 The population averaged map is much closer to the distribution of meiotic crossovers than the CEU map. We calculated and plotted the ratio between the observed and expected numbers of crossovers overlapping hotspots from each of the 20 bins for CEPH (A) and Hutterite (B) crossovers. We estimated expected numbers of crossovers overlapping hotspots for crossovers intervals re-distributed according to the population-averaged or CEU maps.
(0.85 MB TIF)

Figure S12 Estimation of true proportion of crossovers that originate in hotspots. The percentages of crossovers that do not overlap hotspots were calculated for all crossovers and subsets of crossovers mapped to intervals smaller than 20 Kb and 50 Kb. For comparison, the same percentage was calculated for randomly distributed crossovers. Calculations were performed separately for peak-defined CEU hotspots and LDHot-defined hotspots. In addition, we plotted the adjusted percentage of non-predicted crossovers (see Text S1 for details of calculations).
(0.48 MB TIF)

Figure S13 All sets of hotspots predict crossovers better than expected from population-averaged map. We calculated and plotted the observed and expected fraction of crossover intervals smaller than 50 Kb overlapping 10,000 strongest hotspots defined in several ways. We have used LDHot hotspots (LDHot) and peak-based hotspots from population-averaged map (HM) and four population specific maps (CEU, YRI, JPT, and CHB). For ranking, we have used either four population-specific or population-averaged strength estimates. For calculating expected fraction crossovers were re-distributed according to population-averaged map.
(0.72 MB TIF)

Figure S14 Genomic distribution of non-predicted crossovers.
(0.36 MB TIF)

Figure S15 Most Hutterite crossovers not predicted by CEU hotspots overlap hotspots from other populations. The fraction of crossover intervals not predicted by CEU hotspots that overlap hotspots found in YRI, CHB, JPT, or any of the other HapMap PhaseII populations (YRI, CHB, or JPT) is plotted. For comparison, the same fraction of crossovers overlapping hotspots from other populations (mean and 95% CI) is plotted for crossovers re-distributed according to the population-averaged map and randomly distributed crossovers.
(0.19 MB TIF)

Figure S16 CHB-A hotspots overlap more crossovers not predicted by CEU-A hotspots compared to hotspots identified from an identically sized CEU-B sample. We randomly divided 60 individuals from the CEU sample in two sub-samples, CEU-A and CEU-B containing 30 individuals each and an identically sized subset of CHB sample, CHB-A. We then calculated recombination rate maps and identified hotspots on chromosome 6 for each of the 100 samples. (A) The fraction of crossover intervals (mean and 90% CI) not predicted by CEU-A hotspots that overlap hotspots found in CEU-B or CHB-A. (B,C) Histograms of the numbers of chromosome 6 crossover intervals not overlapping CEU-A, CHB-A, CEU-A & CEU-B and CEU-A & CHB-A hotspots. (B) All CEPH crossovers mapped to chromosome 6 (N = 244), (C) Hutterite crossover interavals smaller than 20 Kb mapped to chromosome 6 (N = 189).
(1.94 MB TIF)

Figure S17 The average MAF is not lower in the not-predicted crossover regions compared to that in the predicted crossover regions. We calculated and plotted mean value of minor allele frequency in four population samples for all SNPs located inside crossover intervals.
(0.11 MB TIF)

Table S1 Positions of crossovers mapped in CEPH pedigrees. All coordinates are given relative to NCBI35 and NCBI36 versions of human genome assembly.
(0.50 MB XLS)

Table S2 Summary of the crossover detection simulation.
(0.02 MB XLS)

Table S3 Percentages of genetic and physical map found inside hotspots for each of the four population samples.
(0.02 MB XLS)

Table S4 Summary of CEU bins.
(0.02 MB XLS)

Table S5 Summary of Adjustment calculations.
(0.02 MB XLS)

Text S1 Supplementary methods.
(0.03 MB PDF)
