# Supplementary material for: Genetic Crossovers Are Predicted Accurately by the Computed Human Recombination Map
Source: PLoS Genet. 2010 Jan 29;6(1):e1000831. doi: 10.1371/journal.pgen.1000831 (PMC2813264; doi:10.1371/journal.pgen.1000831)
Supplement: Figure S3 — Summary information on hotspot maps in four HapMap Phase II samples. Histograms of the distributions of the hotspot strength, inter-hotspot distance and hotspot width are shown for CEU (A), YRI (B), CHB (C), and JPT (D) samples. In addition, the figure shows quantiles and mean values calculated for the corresponding distributions. (0.05 MB PDF) [file pgen.1000831.s003.pdf]

A

CEU hotspots

width(Kb)

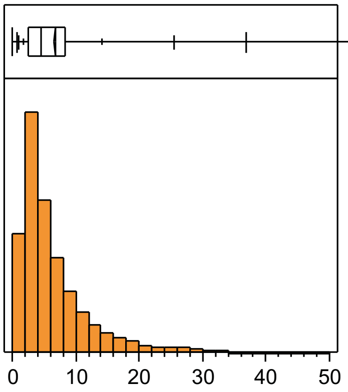

Quantiles

100.0%	maximum	99.876
99.5%		36.844
97.5%		25.419
90.0%		14.252
75.0%	quartile	8.262
50.0%	median	4.570
25.0%	quartile	2.634
10.0%		1.729
2.5%		1.110
0.5%		0.671
0.0%	minimum	0.120

Moments

Mean	6.6920104
Std Dev	6.5275645
Std Err Mean	0.0304774
upper 95% Mean	6.7517465
lower 95% Mean	6.6322743
N	45872

strength(cM)

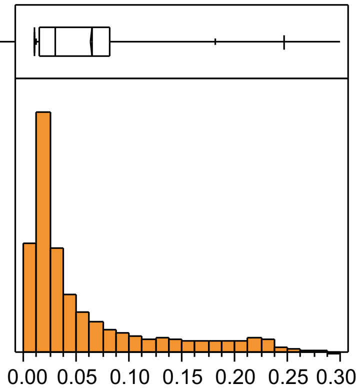

Quantiles

100.0%	maximum	2.7276
99.5%		0.4063
97.5%		0.2464
90.0%		0.1820
75.0%	quartile	0.0819
50.0%	median	0.0300
25.0%	quartile	0.0156
10.0%		0.0117
2.5%		0.0104
0.5%		0.0101
0.0%	minimum	0.0100

Moments

Mean	0.0647455
Std Dev	0.0801971
Std Err Mean	0.0003744
upper 95% Mean	0.0654794
lower 95% Mean	0.0640116
N	45872

distance(Kb)

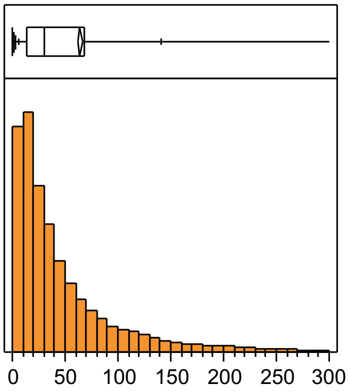

Quantiles

100.0%	maximum	21798
99.5%		650
97.5%		316
90.0%		142
75.0%	quartile	68
50.0%	median	31
25.0%	quartile	14
10.0%		6.764
2.5%		3.62124
0.5%		2.1986
0.0%	minimum	0.6535

Moments

Mean	64.387688
Std Dev	211.38769
Std Err Mean	0.9869743
upper 95% Mean	66.322173
lower 95% Mean	62.453203
N	45872

B

YRI hotspots

width(Kb)

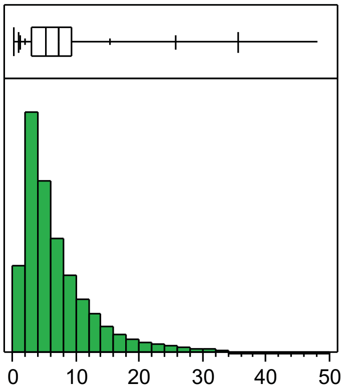

Quantiles

100.0%	maximum	98.019
99.5%		35.669
97.5%		25.825
90.0%		15.306
75.0%	quartile	9.359
50.0%	median	5.276
25.0%	quartile	3.078
10.0%		2.018
2.5%		1.349
0.5%		0.913
0.0%	minimum	0.157

Moments

Mean	7.3675498
Std Dev	6.5437777
Std Err Mean	0.0262974
upper 95% Mean	7.4190928
lower 95% Mean	7.3160068
N	61920

strength(cM)

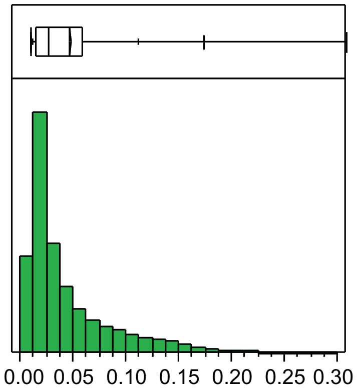

Quantiles

100.0%	maximum	1.1804
99.5%		0.3091
97.5%		0.1743
90.0%		0.1120
75.0%	quartile	0.0596
50.0%	median	0.0276
25.0%	quartile	0.0156
10.0%		0.0118
2.5%		0.0104
0.5%		0.0101
0.0%	minimum	0.0100

Moments

Mean	0.0475548
Std Dev	0.0527357
Std Err Mean	0.0002119
upper 95% Mean	0.0479702
lower 95% Mean	0.0471395
N	61920

distance(Kb)

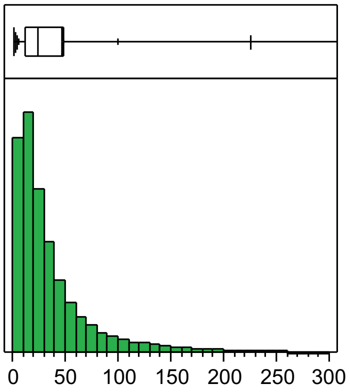

Quantiles

100.0%	maximum	21808
99.5%		480
97.5%		226
90.0%		100
75.0%	quartile	49
50.0%	median	24
25.0%	quartile	12
10.0%		6.5335
2.5%		3.85051
0.5%		2.6008
0.0%	minimum	1

Moments

Mean	47.724378
Std Dev	167.0205
Std Err Mean	0.6712036
upper 95% Mean	49.039938
lower 95% Mean	46.408817
N	61920

C

CHB hotspots

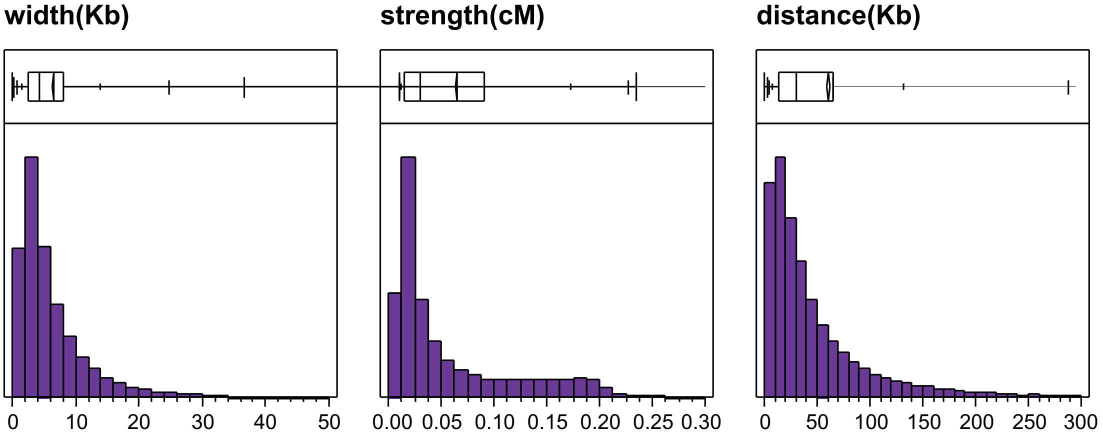

width(Kb)

Quantiles

100.0%	maximum	98.607
99.5%		36.716
97.5%		24.805
90.0%		13.917
75.0%	quartile	8.087
50.0%	median	4.404
25.0%	quartile	2.471
10.0%		1.529
2.5%		0.796
0.5%		0.348
0.0%	minimum	0.102

Moments

Mean	6.4812655
Std Dev	6.4811268
Std Err Mean	0.0294314
upper 95% Mean	6.5389515
lower 95% Mean	6.4235796
N	48493

strength(cM)

Quantiles

100.0%	maximum	3.6678
99.5%		0.4206
97.5%		0.2277
90.0%		0.1721
75.0%	quartile	0.0902
50.0%	median	0.0303
25.0%	quartile	0.0158
10.0%		0.0118
2.5%		0.0104
0.5%		0.0101
0.0%	minimum	0.0100

Moments

Mean	0.0644495
Std Dev	0.0776684
Std Err Mean	0.0003527
upper 95% Mean	0.0651408
lower 95% Mean	0.0637582
N	48493

distance(Kb)

Quantiles

100.0%	maximum	23050
99.5%		578
97.5%		287
90.0%		132
75.0%	quartile	65
50.0%	median	31
25.0%	quartile	14
10.0%		7.2261
2.5%		3.83068
0.5%		2.31697
0.0%	minimum	0.726

Moments

Mean	60.788091
Std Dev	205.23494
Std Err Mean	0.9319911
upper 95% Mean	62.614806
lower 95% Mean	58.961376
N	48493

D

JPT hotspots

width(Kb) **strength(cM)** **distance(Kb)**

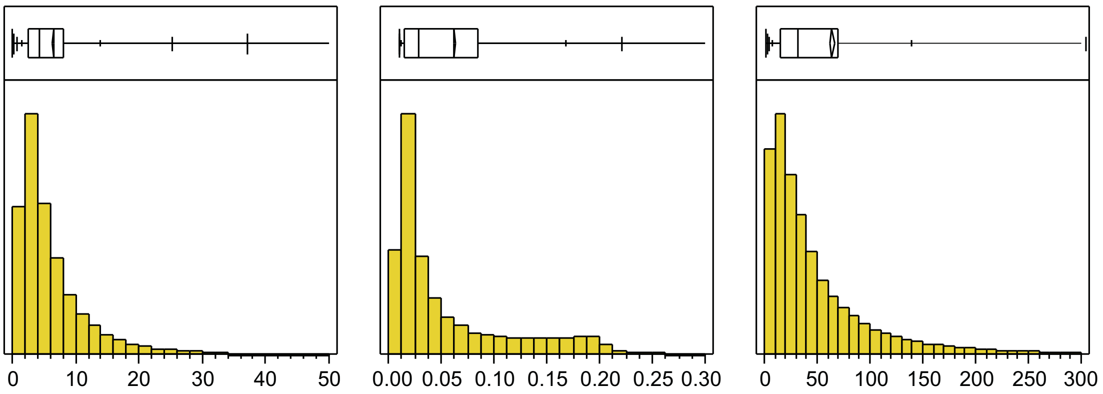

width(Kb)

Quantiles

100.0%	maximum	99.360
99.5%		37.026
97.5%		25.307
90.0%		13.934
75.0%	quartile	8.031
50.0%	median	4.394
25.0%	quartile	2.474
10.0%		1.520
2.5%		0.790
0.5%		0.348
0.0%	minimum	0.102

Moments

Mean	6.4975993
Std Dev	6.528789
Std Err Mean	0.0304861
upper 95% Mean	6.5573525
lower 95% Mean	6.4378461
N	45863

strength(cM)

Quantiles

100.0%	maximum	1.3217
99.5%		0.4022
97.5%		0.2209
90.0%		0.1680
75.0%	quartile	0.0854
50.0%	median	0.0295
25.0%	quartile	0.0157
10.0%		0.0118
2.5%		0.0104
0.5%		0.0101
0.0%	minimum	0.0100

Moments

Mean	0.0623899
Std Dev	0.0733403
Std Err Mean	0.0003425
upper 95% Mean	0.0630612
lower 95% Mean	0.0617187
N	45863

distance(Kb)

Quantiles

100.0%	maximum	21574
99.5%		614
97.5%		305
90.0%		139
75.0%	quartile	69
50.0%	median	33
25.0%	quartile	15
10.0%		7.479
2.5%		3.9479
0.5%		2.43562
0.0%	minimum	0.788

Moments

Mean	64.3225
Std Dev	207.82854
Std Err Mean	0.9704518
upper 95% Mean	66.2246
lower 95% Mean	62.420399
N	45863
