# Supplementary material for: Genetic Crossovers Are Predicted Accurately by the Computed Human Recombination Map
Source: PLoS Genet. 2010 Jan 29;6(1):e1000831. doi: 10.1371/journal.pgen.1000831 (PMC2813264; doi:10.1371/journal.pgen.1000831)

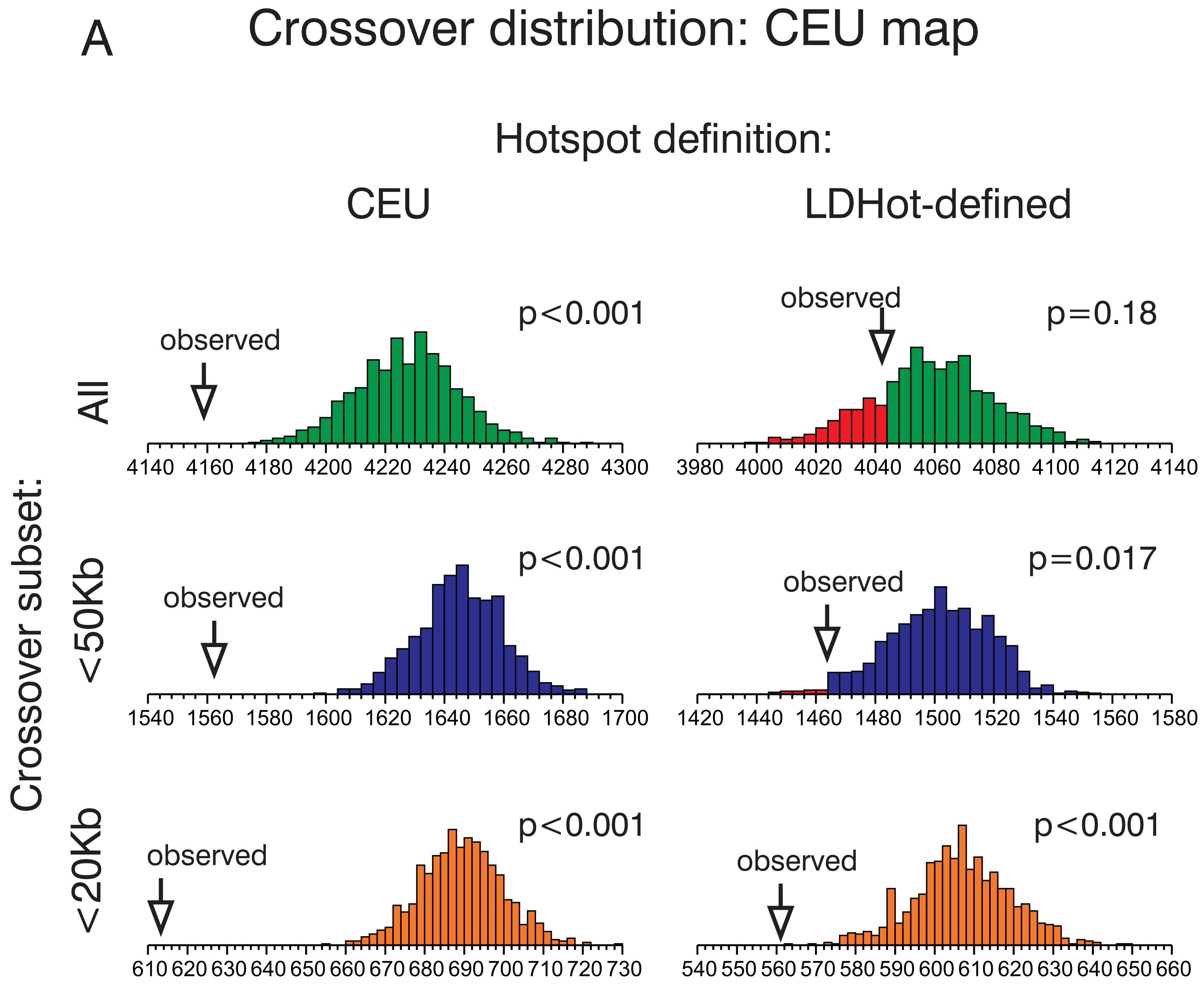
A
Crossover distribution: CEU map
Hotspot definition:
CEU
LDHot-defined
Crossover subset:
All
<50Kb
<20Kb
observed
p<0.001
p=0.18
p<0.001
p=0.017
p<0.001
p<0.001
4140 4160 4180 4200 4220 4240 4260 4280 4300
3980 4000 4020 4040 4060 4080 4100 4120 4140
1540 1560 1580 1600 1620 1640 1660 1680 1700
1420 1440 1460 1480 1500 1520 1540 1560 1580
610 620 630 640 650 660 670 680 690 700 710 720 730
540 550 560 570 580 590 600 610 620 630 640 650 660

Crossover distribution: population-averaged map

B

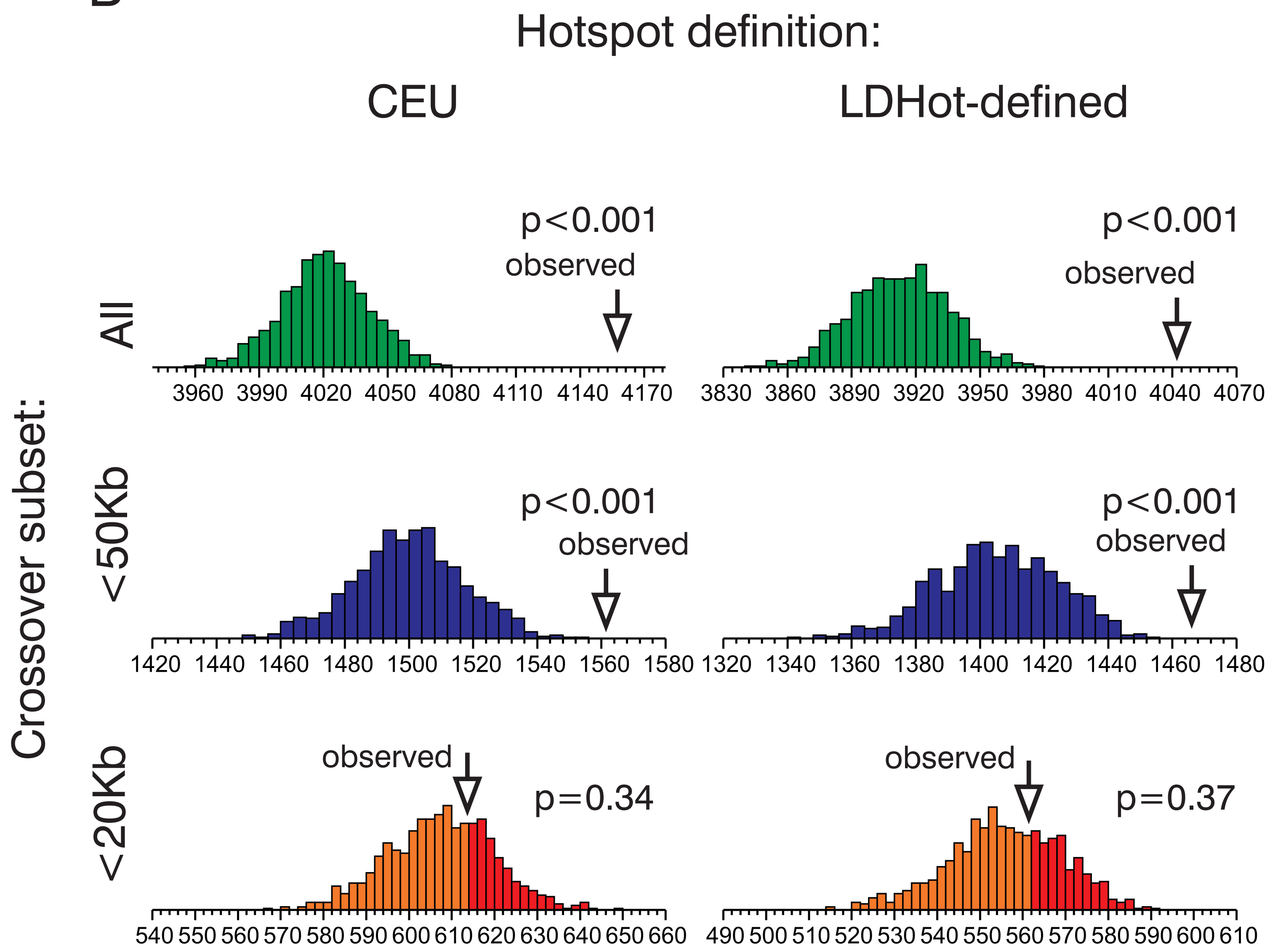

Supplement: Figure S8 — Estimation of the statistical significance of the differences between observed and expected numbers of predicted crossovers. Hotspots predict a significantly smaller number of CEPH crossovers than expected from CEU map (A) and significantly larger number of CEPH crossovers than expected from population-averaged map (B). On the graph the histograms of the expected numbers of crossovers overlapping CEU and LDHot hotspots are plotted (1,000 samples) for three subsets of the crossovers (defined as in text before). For the estimation of expected numbers of predicted crossovers we randomized positions of crossover intervals in the genome according to probabilities determined by CEU (A) and population-averaged (B) recombination rates. The observed numbers of crossovers overlapping CEU or LDHot hotspots for the crossovers mapped in CEPH pedigrees are shown by arrows. The one-sided probability of finding the observed number or fewer of randomly distributed crossovers predicted by hotspots is in the range from 0.001 to 0.20 for crossovers distributed according to CEU map. The one-sided probability of finding the observed number or more of randomly distributed crossovers predicted by hotspots is less than 0.001 for two larger subsets of crossovers distributed according to population-averaged map. (0.03 MB PDF) [file pgen.1000831.s008.pdf]
