# Supplementary figures and images for: Genetic Crossovers Are Predicted Accurately by the Computed Human Recombination Map
Source: PLoS Genet. 2010 Jan 29;6(1):e1000831. doi: 10.1371/journal.pgen.1000831 (PMC2813264; doi:10.1371/journal.pgen.1000831)

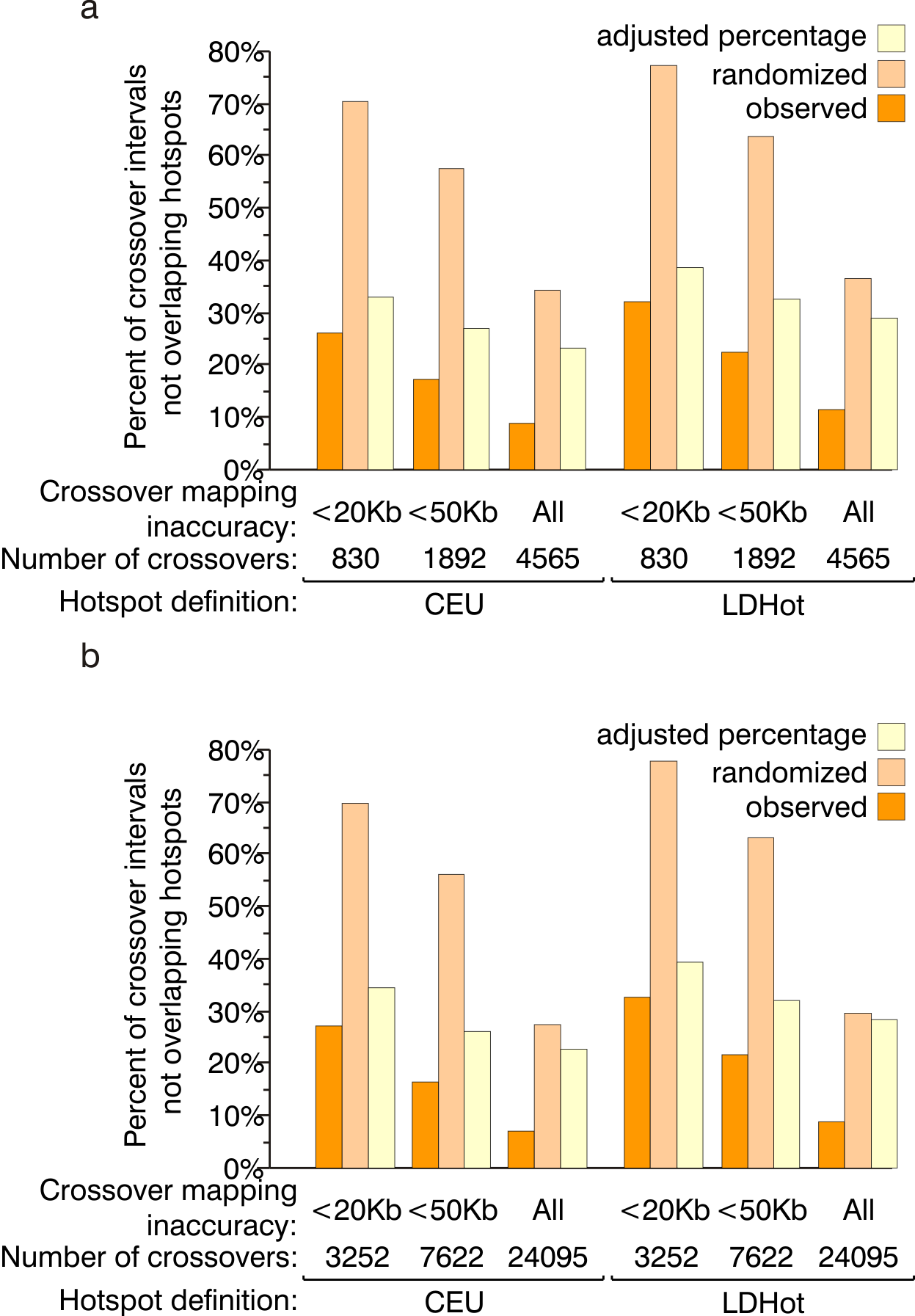

Supplement: Figure S12 — Estimation of true proportion of crossovers that originate in hotspots. The percentages of crossovers that do not overlap hotspots were calculated for all crossovers and subsets of crossovers mapped to intervals smaller than 20 Kb and 50 Kb. For comparison, the same percentage was calculated for randomly distributed crossovers. Calculations were performed separately for peak-defined CEU hotspots and LDHot-defined hotspots. In addition, we plotted the adjusted percentage of non-predicted crossovers (see Text S1 for details of calculations). (0.48 MB TIF) [file pgen.1000831.s012.tif]

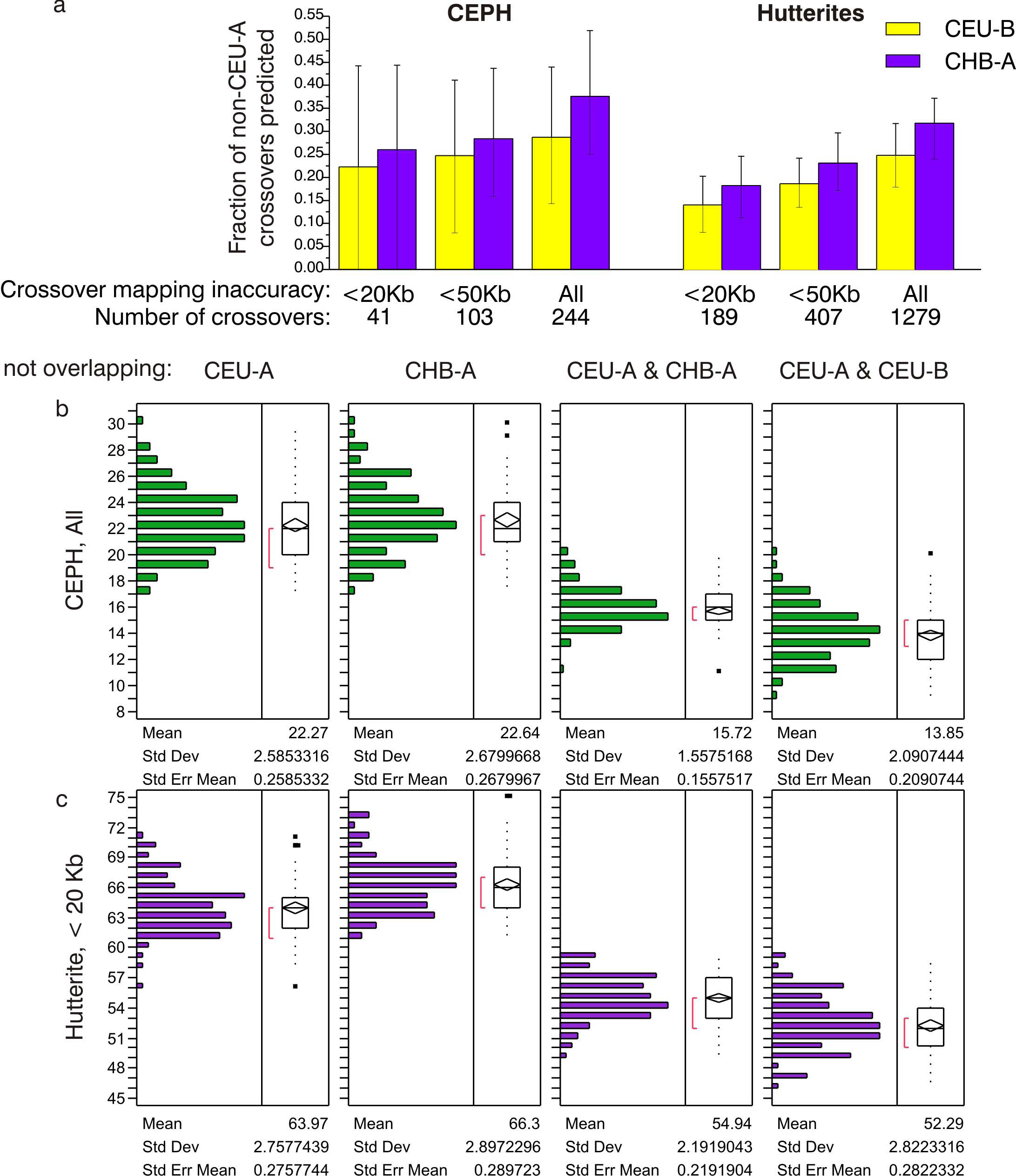

Supplement: Figure S16 — CHB-A hotspots overlap more crossovers not predicted by CEU-A hotspots compared to hotspots identified from an identically sized CEU-B sample. We randomly divided 60 individuals from the CEU sample in two sub-samples, CEU-A and CEU-B containing 30 individuals each and an identically sized subset of CHB sample, CHB-A. We then calculated recombination rate maps and identified hotspots on chromosome 6 for each of the 100 samples. (A) The fraction of crossover intervals (mean and 90% CI) not predicted by CEU-A hotspots that overlap hotspots found in CEU-B or CHB-A. (B,C) Histograms of the numbers of chromosome 6 crossover intervals not overlapping CEU-A, CHB-A, CEU-A & CEU-B and CEU-A & CHB-A hotspots. (B) All CEPH crossovers mapped to chromosome 6 (N = 244), (C) Hutterite crossover interavals smaller than 20 Kb mapped to chromosome 6 (N = 189). (1.94 MB TIF) [file pgen.1000831.s016.tif]
