# Supplementary material for: Genetic Crossovers Are Predicted Accurately by the Computed Human Recombination Map
Source: PLoS Genet. 2010 Jan 29;6(1):e1000831. doi: 10.1371/journal.pgen.1000831 (PMC2813264; doi:10.1371/journal.pgen.1000831)
Supplement: Text S1 — Supplementary methods. (0.03 MB PDF) [file pgen.1000831.s023.pdf]

Supplementary Methods:

Recombination Rate calculations:

To calculate recombination rates we used LDHat version 2[1] with minor modifications introduced to simplify the use of the program in a batch environment. Calculations were for 10^7 iterations, sampled every 5000 iterations and the first 200 observations (10% of all retained observations) were discarded. Data were split in segments of ~5000 SNPs with a 500 SNP overlap between segments. We have used phased haplotypes (release 21a) from complete Phase II data from the HapMap project. All genotype data were downloaded from the HapMap website (www.hapmap.org). For a detailed description of HapMap samples see www.hapmap.org and [2,3]. Calculations were performed for each population sample separately on the NIH Biowulf Linux cluster on 64-130 nodes. To convert population recombination rate ρ into the per-individual rate *r* we used previously determined effective population sizes[2,3].

Hotspot identification:

Briefly, hotspots were defined as narrow peaks in the recombination rate profile (peak width < 50 Kb in 99.9%) with a calculated strength above 0.01 cM. The peak identification algorithm is described in more detail below:

First, we detect initial peaks in the profile as map segments were the first derivative is equal to 0 and the 2nd derivative is negative. Then, for each peak we fit a normal curve to the recombination rate profile in the 50 Kb genome region around the center of the peak. Next, we refine peak boundaries. First we define the maximum region containing a peak. The maximum region width was defined as the smaller of 2×fitted peak width (FWHM obtained from curve fitting) or 50 Kb. We include in the analysis all map segments overlapping the maximum region containing the peak. The peak boundaries were extended to include all map segments with recombination rates above mean recombination rate inside the maximum region containing peak. The procedure was repeated for each of the initially detected peaks. Next, we re-analyzed all of the defined peaks to correct for overlap. If overlaps were detected, peak boundaries were set in the valley between peaks. Then we applied post-filtering procedures aimed to remove peaks that do not overlap the initial peak after the fitting The resultant well-defined peaks were further filtered to include only peaks without extended flat regions on the top (peak width <100 Kb). Less than ~0.04% of all peaks have such extended flat regions >100 Kb in lengths. The use of data at higher resolution improves the performance of this procedure. The Perl/PDL code used to extract peaks from recombination rate profiles is available upon request. In this definition of hotspots we do not attempt to estimate the statistical significance of the presence of the peak relying on the accurate reconstruction of recombination rate maps instead.

The hotspot is described by its peak position, width and strength (area of the peak or total genetic length or % of recombination occurring inside a given hotspot per meiotic division). The number of hotspots identified ranges from 45,863 in the JPT sample up to 61,920 in the YRI sample, which is consistent with previous estimates[2,4] (Figure S3). Total number of all peaks including weaker hotspots with an estimated strength below 0.01 and very wide peaks ranges from 140,378 to 182,994. These numbers represent upper limit for number of hotspots that can be detected as peaks in HapMap Phase II data. The mean distance between hotspots is around 50-60 Kb, the mean hotspot width is

approximately 6.5-7 Kb and the mean strength is approximately 0.05-0.07 cM (Figure S3). Hotspots account for 77% of the total genetic map length in CEU sample and cover 10.5% of the genome (see Table S3, Figure S3).

Crossover mapping:
DNA samples were obtained from the Coriell cell repository. Samples were genotyped using the Affymetrix 500K array set according to the recommendations of the manufacturer. All genotypes were determined by the BRLMM algorithm. We genotyped only siblings from CEPH reference pedigrees 1334, 1340, 1341, 1350, 1362, 1408, 1420, 1447, 1454 and 1459. Previously determined parental genotypes were downloaded from the Affymetrix web site (http://www.affymetrix.com/support/technical/sample_data/500k_hapmap_genotype_data.affx). We downloaded genotypes for DNA samples NA10846, NA10847 (parents from CEPH/UTAH Pedigree 1334), NA07019, NA07029 (parents from CEPH/UTAH Pedigree 1340), NA7048, NA6991 (parents from CEPH/UTAH Pedigree 1341), NA10855, NA10856 (parents from CEPH/UTAH Pedigree 1350), NA10860, NA10861 (parents from CEPH/UTAH Pedigree 1362), NA10830, NA10831 (parents from CEPH/UTAH Pedigree 1408), NA10838, NA10839 (parents from CEPH/UTAH Pedigree 1420), NA12752, NA12753 (parents from CEPH/UTAH Pedigree 1447), NA12801, NA12802 (parents from CEPH/UTAH Pedigree 1454) and NA12864, NA12865 (parents from CEPH/UTAH Pedigree 1459). These samples are part of the CEU sample of the HapMap project and are also available from HapMap website (www.hapmap.org). We genotyped DNA samples NA12138, NA12139, NA12141, NA12238, NA12143 (CEPH/UTAH Pedigree 1334), NA07062, NA07053, NA07008, NA07040, NA07342, NA07027, NA11821 (CEPH/UTAH Pedigree 1340), NA07343, NA07044, NA07012, NA07344, NA07021, NA07006, NA07010, NA07020 (CEPH/UTAH Pedigree 1341), NA11822, NA11824, NA11825, NA11826, NA11827, NA11828 (CEPH/UTAH Pedigree 1350), NA11982, NA11983, NA11984, NA11985, NA11986, NA11987, NA11988, NA11989, NA11990, NA11991, NA11996 (CEPH/UTAH Pedigree 1362), NA12147, NA12148, NA12149, NA12150, NA12151, NA12152, NA12153, NA12157 (CEPH/UTAH Pedigree 1408), NA11997, NA11998, NA11999, NA12000, NA12001, NA12002, NA12007 (CEPH/UTAH Pedigree 1420), NA12754, NA12755, NA12756, NA12757, NA12758, NA12759, NA12764, NA12765 (CEPH/UTAH Pedigree 1447), NA12803, NA12804, NA12805, NA12806, NA12807, NA12808, NA12809, NA12810, NA12811, NA12816 (CEPH/UTAH Pedigree 1454), NA12866, NA12867, NA12868, NA12869, NA12870, NA12871, NA12876 (CEPH/UTAH Pedigree 1459).

To map crossovers with the maximum possible precision we used a multi-step algorithm briefly outlined below. We first phase all of the SNPs (both heterozygous in one of the parents and heterozygous in both parents) and only then call crossovers. The need for the development of specialized approaches arises from the relatively high error rate of SNP genotyping on arrays and the inability to phase chromosomes based on single SNP markers.

First, we phased genotypes based on mendelian inheritance patterns in places where trivial phase inference is possible. Such positions are all homozygous positions and those heterozygous positions where one of the parental genotypes is homozygous. Then we

inferred haplotype in the heterozygous positions where both parental genotypes are heterozygous using a combinatorial approach. The approach is based on the conservation of the relative haplotype configuration for extended regions. That is, if AAABA is the known haplotype configuration of five siblings determined at the positions where phase inference is trivial, compatible haplotype configurations in positions nearby (implying that there was no crossover) will be AAABA and BBBAB. In the same manner we can determine two compatible haplotype configurations derived from the other parent. Next, we can combine all compatible haplotype configurations of both origins and determine ones that give a known genotype. In most cases there will be only one compatible configuration, even in the presence of a moderate level of missing genotypes. This approach can only be used if the number of siblings is greater than 2. We genotyped between 5 and 11 siblings per family. Next we first roughly identified and then refined positions of crossovers. We performed multiple rounds of correction for possible genotyping errors and for potential aberrant chromosome structures such as extended regions of homozygosity. Finally, we excluded all double crossovers determined to be within 1 Mb of each other due to their higher likelihood of being caused by genotyping errors. In our work we focus on the global correlation between positions of crossovers with calculated hotspots and such exclusion does not substantially biases the results. Assuming that there are no crossover interference and crossovers are distributed according to the population-averaged map, we can expect around 4% such tight double crossovers (Table S2). In our CEPH data we had mapped 12.5% of all crossovers within 1 Mb of each other. Due to the highly redundant nature of the data, the algorithm is resistant to relatively high genotyping error level and to missing data (~10%).

To prove the correctness of our algorithm we performed simulation. First, we phased parental genotypes in all CEPH pedigrees. We then re-distributed all crossovers according to the population averaged map and generated genotypes with defined crossover positions. Then we mapped crossovers using our algorithm. We ran simulation 100 times. We correctly identify the positions of 95% of all crossovers (Table S2). Out of the 5% not mapped crossovers, the majority (3.8%) are from tight "double-crossovers" that are intentionally filtered and the remaining 1.3% are located too close to the ends of chromosomes to be identified.

The crossovers were originally mapped in the NCBI36 coordinates and then re-mapped onto the coordinates relative to the NCBI35 reference genome assembly version using the batch conversion tool at the UCSC genome browser website (http://genome.ucsc.edu/cgi-bin/hgLiftOver).

The uncertainty in defining crossover positions ranges from 50 bp to over 30 Mb with a median of ~70 Kb (Figure S1, Table S1). The patterns of the distribution of crossovers are consistent with previously reported observations. In particular, we find that male recombination events are biased toward telomeres[5] (Figure S2) and, in agreement with a higher recombination rate in females[5], there is an approximately 60% excess of maternal crossovers over paternal crossovers (2934 maternal crossovers compared to 1844 paternal crossovers). To allow direct comparison between Hutterite and CEPH data we excluded from the analysis crossovers mapped to chromosome X.

Randomization of crossover positions:

To investigate the relationship between crossovers and hotspots and to estimate the fraction of crossover intervals that overlap hotspots by chance we randomized the positions of crossover intervals in the genome. We used two simulation approaches. In one approach to randomize positions of crossovers while preserving some of the observed large scale non-uniformity in crossover distribution we uniformly re-distributed crossovers within genome near their original locations ("regionally-biased" randomization). We preserved the chromosomal origin of each crossover region but randomly shifted it from its original position. The random offset ranged from 200 Kb to 1.2 Mb. In this randomization strategy we preserve large-scale features of the distribution of crossovers in the genome, such as the excess of paternal crossovers near telomeres and the non-equal recombination rates on different chromosomes, but disrupt the fine-scale relationship between crossovers and hotspots.

In the second approach we distributed crossovers in the genome according to the recombination rate map. As in "regionally-biased" randomization we preserved chromosomal origin of each crossover region but randomly placed center of with the probabilities defined by the calculated recombination rate map. That is, the probability of finding the center of the crossover interval in the region with high recombination rate is high and in the region with low recombination rate is low. We generated random sets of crossover intervals preserving both the sizes and number of crossovers (4565). When we studied the relationship between the crossover mapping inaccuracy and the fraction of predicted crossovers, crossovers sizes were set to a predetermined value. The number of crossovers was still the same (4565).

Because in our randomization strategy we do not take into account details of the distribution of SNPs on 500K arrays, there is a potential to introduce bias in our calculations. Our mapping will always have crossover interval ends at SNPs which is not true for the randomization strategy outlined above. We verified our approach by performing detailed simulation.

We simulated whole XO detection and downstream analysis (see above and Table S2). We generated 100 sets where crossovers were distributed according to the population-averaged map. We then, similarly to the analysis presented on Figure 1, calculated the expected fraction of predicted crossovers and compared it to the observed fraction of predicted crossovers (Figure S6). We again find that the expected fraction of predicted crossovers agrees with the observations. Thus, we confirm our major conclusion using a more detailed simulation.

This approach more detailed simulation approach depends on the availability of phased genotypes and is therefore suitable only for the CEPH data. Thus, to allow direct comparison of CEPH and Hutterite data we used less detailed simulation outlined above for all analyses except for the calculations presented on Figure S6.

Adjustments of the percentage of crossover intervals overlapping hotspots and estimation of the fraction of crossovers that overlap hotspots by chance:

The inaccuracy of crossover mapping is rather high and comparable with inter-hotspot distance. Thus, a substantial fraction of crossovers that originated outside hotspots might have been mapped to intervals that overlap hotspots due to the inaccuracy of the mapping. To address this issue we analyzed separately several subsets of crossover

intervals of different size and performed computer modeling by randomly distributing crossover intervals within the genome. The adjustment procedure for calculating correct fraction of crossovers that originated in hotspots was as follows:
Let's assume that we have two fractions of crossovers – crossovers that originated in hotspots and crossovers that are randomly distributed in the genome. If $X_{hotspots}$ is the relative proportion of crossovers that originated in hotspots and X_{random} is the relative proportion of randomly distributed crossovers, the observed fraction of crossover intervals overlapping hotspots $F_{observed}^{overlapping}$ may be expressed as:

$$F_{observed}^{overlapping} = F_{hotspots}^{overlapping} \times X_{hotspots} + F_{random}^{overlapping} \times X_{random} \quad \textbf{(1)},$$

where $F_{hotspots}^{overlapping}$ is the fraction of crossovers that originated in hotspots that overlap hotspots and $F_{random}^{overlapping}$ is the fraction of randomly distributed crossovers that overlap hotspots. We are interested in finding the relative proportion of randomly distributed crossovers X_{random} from the observed fraction of crossover intervals overlapping hotspots $F_{observed}^{overlapping}$.

Obviously, $F_{hotspots}^{overlapping} = 1$ and $X_{hotspots} + X_{random} = 1$ (**2**)
By solving equations (**1**) and (**2**) we find that:

$$X_{random} = \frac{(1 - F_{observed}^{overlapping})}{(1 - F_{random}^{overlapping})} \quad \textbf{(3)}$$

A subset of randomly distributed crossovers originates, however, from inside the hotspots. In the case of uniformly distributed crossovers we can simply account for that random overlap by multiplying the estimated fraction of randomly distributed crossovers by the fraction of the genomic sequence outside hotspots. Thus,

$$F_{adjusted}^{non-overlapping} = X_{random} \times (1 - G_{hotspots}) \ \textbf{(4)} \text{ or } F_{adjusted}^{non-overlapping} = \frac{F_{observed}^{non-overlapping}}{F_{random}^{non-overlapping}} \times (1 - G_{hotspots})$$

(**5**) where $F_{observed}^{non-overlapping} = (1 - F_{observed}^{overlapping})$ is the observed fraction of crossover intervals not overlapping hotspots, $F_{random}^{non-overlapping} = (1 - F_{random}^{overlapping})$ is the fraction of randomly distributed crossover intervals not overlapping hotspots and $G_{hotspots}$ is the proportion of genomic sequence inside hotspots.

In the equation (1) $F_{random}^{overlapping} \times X_{random} = F_{random}^{overlapping} \times \frac{(1 - F_{observed}^{overlapping})}{(1 - F_{random}^{overlapping})}$ is the fraction of crossovers that overlap hotspots by chance. On Figure 2 we plotted this value.

The fraction of randomly distributed crossovers overlapping hotspots $F_{random}^{non-overlapping}$ was estimated from a simulation (see below) and the fraction of genomic sequence inside hotspots $G_{hotspots}$ was determined previously and is given in Table S2. Results of applying adjustment to CEPH and Hutterite crossovers are presented on Figuret S12 and

Table S5. We estimate that 23-34% of crossovers originate outside CEU hotspots and 28-39% originate outside LDHot-defined hotspots.

Code availability:

Perl code for pre- and postprocessing of the HapMap data for the recombination rate calculations and constructions of maps at different resolutions, generation of permuted samples, hotspot identification, hotspot map comparisons, crossover mapping from genotype data and random crossover modeling and data summation are available upon request. R, JMP and batch scripts used for statistical analysis are available as well.

Supplementary References:

1. McVean GA, Myers SR, Hunt S, Deloukas P, Bentley DR, et al. (2004) The fine-scale structure of recombination rate variation in the human genome. Science 304: 581-584.
2. (2005) The International HapMap Consortium: A haplotype map of the human genome. Nature 437: 1299-1320.
3. Frazer KA, Ballinger DG, Cox DR, Hinds DA, Stuve LL, et al. (2007) A second generation human haplotype map of over 3.1 million SNPs. Nature 449: 851-861.
4. Myers S, Bottolo L, Freeman C, McVean G, Donnelly P (2005) A Fine-Scale Map of Recombination Rates and Hotspots Across the Human Genome. Science 310: 321-324.
5. Lynn A, Ashley T, Hassold T (2004) Variation in human meiotic recombination. Annu Rev Genomics Hum Genet 5: 317-349.
